# Allometry of the Duration of Flight Feather Molt in Birds

**DOI:** 10.1371/journal.pbio.1000132

**Published:** 2009-06-16

**Authors:** Sievert Rohwer, Robert E. Ricklefs, Vanya G. Rohwer, Michelle M. Copple

**Affiliations:** 1 Burke Museum and Department of Biology, University of Washington, Seattle, Washington, United States of America; 2 Department of Biology, University of Missouri-St. Louis, St. Louis, Missouri, United States of America; Lund University, Sweden

## Abstract

We used allometric scaling to explain why the regular replacement of the primary flight feathers requires disproportionately more time for large birds. Primary growth rate scales to mass (*M*) as *M* ^0.171^, whereas the summed length of the primaries scales almost twice as fast (*M* ^0.316^). The ratio of length (mm) to rate (mm/day), which would be the time needed to replace all the primaries one by one, increases as the 0.14 power of mass (*M* ^0.316^/*M* ^0.171^ = *M* ^0.145^), illustrating why the time required to replace the primaries is so important to life history evolution in large birds. Smaller birds generally replace all their flight feathers annually, but larger birds that fly while renewing their primaries often extend the primary molt over two or more years. Most flying birds exhibit one of three fundamentally different modes of primary replacement, and the size distributions of birds associated with these replacement modes suggest that birds that replace their primaries in a single wave of molt cannot approach the size of the largest flying birds without first transitioning to a more complex mode of primary replacement. Finally, we propose two models that could account for the 1/6 power allometry between feather growth rate and body mass, both based on a length-to-surface relationship that transforms the linear, cylindrical growing region responsible for producing feather tissue into an essentially two-dimensional structure. These allometric relationships offer a general explanation for flight feather replacement requiring disproportionately more time for large birds.

## Abstract 2

Replacement of flight feathers takes disproportionately more time for large birds than it does for small birds, because feather length increases with body size almost twice as fast as feather growth rate increases.

## Author Summary

The pace of life varies with body size and is generally slower among larger organisms. Larger size creates opportunities but also establishes constraints on time-dependent processes. Flying birds depend on large wing feathers that deteriorate over time and must be replaced through molting. The lengths of flight feathers increase as the 1/3 power of body mass, as one expects for a length-to-volume ratio. However, feather growth rate increases as only the 1/6 power of body mass, possibly because a two-dimensional feather is produced by a one-dimensional growing region. The longer time required to grow a longer feather constrains the way in which birds molt, because partially grown feathers reduce flight efficiency. Small birds quickly replace their flight feathers, often growing several feathers at a time in each wing. Larger species either prolong molt over two or more years, adopt complex patterns of multiple feather replacement to minimize gaps in the flight surface, or, among species that do not rely on flight for feeding, simultaneously molt all their flight feathers. We speculate that the extinct 70-kg raptor, *Argentavis magnificens*, must have undergone such a simultaneous molt, living off fat reserves for the duration.

## Introduction

Flight feather molt is a time-demanding activity in the avian annual cycle [1]–[3]. Yet, annual or alternate-year replacement of flight feathers is essential, because physical abrasion and ultraviolet light rapidly degrade even the most sturdy wing quills after two years of use [4],[5]. Because flight performance declines during molt as new feathers are growing [6]–[8], most birds do not overlap molt and breeding, and those that do overlap these activities replace few flight feathers at a time, presumably to minimize the energetic and flight-performance costs of molting on reproduction [5],[9]. Overlap of molt and breeding may be more common in larger species because the time required to rear young, as well as the time required to replace flight feathers, increases with body size [3],[10],[11].

Most smaller birds (i.e., generally <1 kg) replace all their flight feathers annually, and a few do so twice a year [12]–[14]. In contrast, many larger birds (>3 kg) that depend on flight for feeding during the molt shed only a part of their flight feathers annually [5],[15] and require two, and sometimes three, years to complete the molt [16],[17]. For example, no albatross regularly replaces all of its flight feathers in a single bout of molting [18],[19], and the largest albatrosses (*Diomedea exulans* and *D. epomophora*), whose masses reach 10 kg, avoid reproducing during years following successful breeding because of the competing time and resource demands of reproduction and molt.

Although ornithologists have been aware of the protracted molts of large birds for many years, no general argument has been proposed to account for the increased time required for flight feather replacement. To the best of our knowledge, we show for the first time how the allometric scaling of flight feather length and flight feather growth rate with body mass sets an upper limit to complete annual replacement of the primaries at a body mass of about 3 kg. Because feather growth rates do not differ between similarly sized species exhibiting simultaneous versus sequential replacement of the primaries, the resource and energy demands of molting cannot explain why primary growth rate fails to increase with mass as fast as primary length. Rather, we suggest that the architecture of a two-dimensional structure emerging from an essentially one-dimensional follicle constrains the rate of feather growth to slow relative to increasing feather length in larger birds. Finally, these allometric relationships prompt us to ask how the 70-kg raptor, *Argentavis magnificens*, a flying teratorn from the Miocene of Argentina [20],[21], could have organized the replacement of its enormous flight feathers to have had sufficient time also to reproduce.

## Results

### Molt Allometries

**Figure 1:**
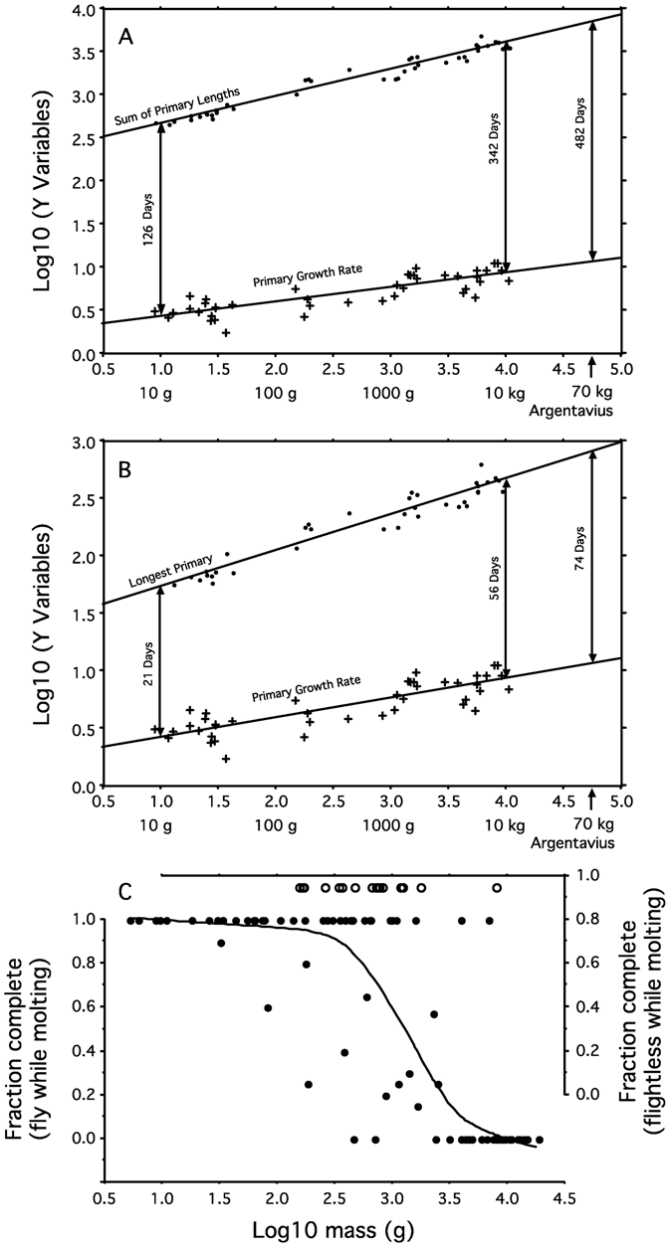
Relationships between mass and flight feather growth rate, length of flight feathers, and completeness of flight feather molts. (A) Allometric relationships between log_10_ mass (g) and log_10_ primary growth rate (mm/d) and log_10_ summed primary length (mm). Arrows indicate the estimated time required to replace all primaries if they are grown one feather at a time for birds of 10 g, 10 kg, and for the extinct teratorn that weighed 70 kg. (B) Same as (A), except primary length is the longest primary. Arrows indicate the estimated time required to replace all the flight feathers in a simultaneous replacement of the primaries. (C) Fraction of individuals (*n* = 20 for most species, see Table S1) showing complete replacement of their primaries in their last molt, plotted against log_10_ mass for 77 species that fly while molting (black dots and loess curve), and the same relationship for 17 species that replace their flight feathers simultaneously (open circles).

Primaries are the longest flight feathers of the wing, technically defined as the quills that attach to the bones of the hand. Most extant birds have 9 or 10 functional primaries [5]. We used allometric scaling to explain the basis for time constraints on primary replacement in the life histories of large birds. We have related primary growth rate (*K*, from the literature, defined as the daily increase in length of individual primaries) and both length of the longest primary and summed length of all the primaries (*L*, from museum specimens) to body mass (*M*) across a wide size-range of birds (masses from [22]) by the allometric function *Y* = a*M* ^b^, where a is a scaling constant and b is the power of the relationship of *Y* to mass. Primary growth rate scales as *M* ^0.171^ (Figure 1A), close to a value of *M* ^0.19^ found by Hedenstrom [3] using other data and assumptions, whereas the combined lengths of all the primary flight feathers (as well as the length of the longest primary) increases with body mass almost twice as fast, as *M* ^0.316^ (Figure 1A). The ratio of length (mm) to rate (mm/day), which is the time required to replace all the feathers one at a time (days), and which is also proportional to the time required to grow the longest primary (Figure 1B), increases as the 0.14 power of mass (*M* ^0.316^/*M* ^0.171^ = *M* ^0.145^). This illustrates why molt is so time consuming for large birds. These scaling relationships set upper and lower limits to the time that birds of different size would need to replace their primaries. Figure 1A approximates the upper time required for molt by assuming the primaries are grown one feather at a time, and Figure 1B approximates the lower limit when all primaries are lost and re-grown at the same time. Of course the actual duration of primary molts varies between these extremes by a factor of close to 10, depending on the number of primaries grown simultaneously.

Birds that fly while molting usually grow only two or three primaries on each wing at the same time. For example, rough-winged swallows, *Stelgidopteryx serripennis*, are 15.9-g aerial foragers that replace an average of only 1.8 primaries at a time, because they forage on the wing while molting [23]. For a 15.9-g bird, the allometric relationships in Figure 1A predict that replacing the nine primaries, one feather at a time, would take 190 days. Adjusting for the number of primaries grown simultaneously reduces this estimate to 105.5 days, which closely matches empirical observations [23].

**Figure 2:**
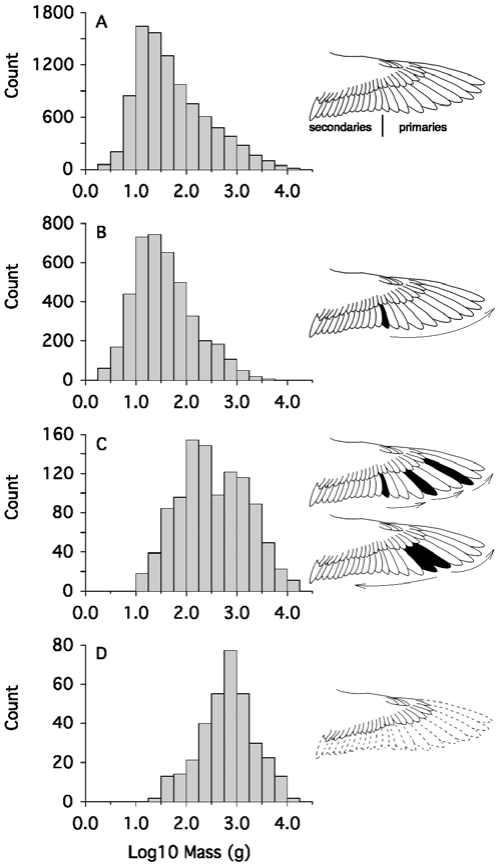
Body size distributions associated with different patterns of primary replacement; dark feathers are points of molt initiation. (A) Size distribution for all flying birds (wing shows primary and secondary flight feathers). (B) Size distribution for species with simple molts, characterized by a single wave of primary replacement. (C) Size distribution for species with complex molts that feature either (1) multiple waves of primary replacement proceeding distally through the primaries, or (2) division of the primaries into two molt series with feathers replaced in opposite directions. (D) Size distribution for species that replace their primaries (and secondaries) simultaneously (wing shows lost primaries and secondaries depicted by dotted lines). Most species with simple molts (B) fail to approach 15 kg, the approximate upper size limit of birds set by the power available for flight because such large birds would require too much time to replace their flight feathers. But species with complex (C) and, especially, simultaneous primary replacement (D) reach the upper size limit of birds set by the power required for flight because these modes of primary replacement require less time than simple molts.

Simultaneous replacement of the flight feathers is characteristic of many water birds (loons, grebes, waterfowl, many rails, and some alcids) that can swim and dive to forage and escape predators while flightless [24]. In Figure 2B, the distance between the allometric relationships of the length of the longest primary (*M* ^0.313^) and of primary growth rate (as in Figure 1A, *M* ^0.171^) estimates the time (*M* ^0.142^) that simultaneous replacement of the primaries would render an individual flightless. In most forms of simultaneous primary replacement, secondary flight feathers (shorter flight feathers, proximal to the primaries) are replaced at the same time as the primaries, so the full period of flightlessness corresponds to the time required to replace the longest primary—estimated to be 57 days, and observed to be 63 days, for 11.8-kg mute swans *Cygnus olor.* The actual period of flightlessness is somewhat less, because individuals regain flight a few days before the longest primaries are fully grown [25].

### Molt Allometries and Incomplete Molts

For birds that continue to fly while molting, the diverging allometric curves of Figure 1A illustrate how the time required to replace the primaries one by one increases dramatically with body size. Large birds that continue to fly while molting reduce the time spent replacing primaries both by growing several primaries simultaneously and by retaining individual feathers for two or, rarely, even three years [2],[17], but they still spend an ever-increasing fraction of the annual cycle replacing flight feathers. Figure 1C illustrates the body size–dependence of the shift from complete to incomplete primary molts. Most individuals of species with masses below 1 kg replace all of their primaries annually, whereas most individuals of species with masses over 3 kg spread the primary molt over two or more years (Figure 1C). The broad size range for this transition reflects special circumstances for many species. For example, numerous small owls have incomplete molts, possibly because the flight feathers of these nocturnal birds suffer little degradation from ultraviolet light [4] and can be used for more than one year. Some very large birds replace all of their flight feathers every year because they overlap molt and breeding [10],[11] or because they molt for many months. Male wild turkeys (*Meleagris gallopavo*) do not participate in parental care and so they can replace primary feathers for six months each year, beginning well before females [26].

### Primary Replacement and the Maximum Size of Flying Birds

Early theoretical analyses suggested that the size of birds with sustained flapping flight would be limited by the power required for flight, which increases as *M* ^7/6^, and the power available for flight, which increases as *M* ^3/4^ [27],[28]. If these curves actually crossed at about 15 kg, the theory might explain the size of the largest swans and pelicans with sustained flapping flight. Recent analyses by Chattergee and colleagues [21],[29] applying helicopter streamtube theory have confirmed this suggestion, finding that the upper limit of sustained powered flight for birds and pterosaurs is about 15 kg.

We explored whether flight feather replacement might additionally constrain body size evolution in flying birds by considering size distributions of species using each of the three fundamental modes of primary replacement. Patterns of primary replacement have been described for many birds; further, most birds replace the secondary flight feathers during the primary molt, so secondary replacement does not add to the time spent molting flight feathers [12],[16],[30]. Thus, analyses of size constraints based on primary replacement patterns should be general to most birds.

We made three predictions. First, the mode of the size distribution for birds with simple molts (species with a single wave of primary replacement) should be small and the right tail of this distribution should fail to approach the 15-kg limit for powered flight [27],[28]. When the primaries are replaced in a single wave, the only way to reduce the time in molt is to grow more feathers simultaneously. However, the resulting large gaps in the primaries would be detrimental to flight [7],[8], especially in large species that are heavily wing-loaded [31]. Thus large size and simple primary molts should be incompatible for most species.

Second, the size distribution of birds with complex modes of primary replacement should be larger than that for birds with simple primary replacement. Complex molts generate multiple waves of feather replacement (either by stepwise molts or by dividing the primaries into at least two replacement groups). Complex primary molts allow more feathers to be replaced at once, and also reduce the size of gaps in the wing surface because adjacent primary feathers partially overlap each other [32]. Because complex molts reduce time constraints on molting by maximizing the number of feathers growing simultaneously, compared with loss in wing area, the modal size of species with complex molts should exceed that for species with simple molts; further, the right tail of this distribution could extend towards the upper size limit for powered flight of about 15 kg [27],[28].

Third, species with simultaneous flight feather replacement must be constrained least by the time required to replace their flight feathers (Figure 1B). Hence their size distribution should exhibit the highest mode, and its right tail should extend to 15 kg. This prediction assumes that species that molt simultaneously can meet the energetic and nutritional demands of growing their many flight feathers simultaneously and, thus, reduce the time required to replace all their flight feathers to the time needed to grow their longest primary (Figure 1B). The observation that feather growth rates do not differ between species with simultaneous and sequential replacement of the primaries (see below) supports this assumption. Because replacing the flight feathers simultaneously is so time-efficient, simultaneous replacement of the wing quills should also be favored in small aquatic species that can safely undergo a period of flightlessness, possibly giving the body size distribution for simultaneous molters a left skew.

### Primary Replacement Strategy and Body Size Distributions: Results

Figure 2 presents four sets of size distributions with cartoons illustrating the primary and secondary flight feathers (Figure 2A) and the three fundamental modes of primary replacement (Figure 2B–2D). The distribution of log-transformed body masses for all flying birds regardless of molt strategy (Figure 2A, masses from [22]) exhibits a relatively small modal size (∼13 g) and a strong right skew (*g_1_* = 0.794, *p*<0.0001, *n* = 9,324), which characterizes size distributions for most animals [33],[34].

Among species with simple molts, log_10_(*M*) (mode = 24 g, Figure 2B) is also strongly right-skewed (*g_1_* = 0.634, *p*<0.0001, *n* = 4,163), but the extreme right tail of this distribution falls short of the upper size limit of contemporary flying birds (15 kg). Species with complex molts are much larger than those with simple molts (Mann Whitney *p*<0.0001), having a modal body mass of 133 g and a right tail that reaches the size of the largest flying birds (Figure 2C). The size distribution of species with complex molts is not significantly skewed (*g_1_* = 0.096, *p*>0.20, *n* = 1,043), as predicted, presumably because the right tail is constrained by the power requirements for flight [27],[28] and because the left tail is drawn out by numerous small tropical species that have complex modes of primary replacement to increase breeding frequency [32]. That complex molts permit larger body sizes than simple molts suggests that, if birds must fly while molting, a transition to one of the two complex modes of primary replacement is prerequisite to evolving body sizes that approach 15 kg.

Species with simultaneous primary replacement crowd the maximum size of flying birds (Figure 2D) and do so even more strongly than those with complex primary molts (Figure 2C). At 750 g, the modal size for species that molt simultaneously significantly exceeds that of species having both simple and complex molts (Mann-Whitney *p*<0.001 for both simple and complex molts). The size distribution associated with simultaneous primary molts is slightly, but not significantly, left skewed (Figure 2D; *g_1_* = −0.201, *p*>0.10, *n* = 344), presumably because the power requirements for flight sets an upper size limit [27],[28], constraining skew to the left tail of this distribution. The left skew is generated by small species—such as dippers, small alcids, and small rails—with safe molting sites that permit temporary flightlessness. Simultaneous flight feather molts should be favored in these small species for several reasons. First, simultaneous molts are always complete (Figure 1C), eliminating replacement asymmetries that have fitness costs [35]. Second, no developmental organization is required to maintain symmetry in flight feather replacement during simultaneous molt. Third, simultaneous replacement of the primaries minimizes time conflicts between molt and breeding. Finally, simultaneous flight feather molts may be particularly energy efficient if feathers that do not suffer the strain of use while growing can be grown with less cost [36]; we know of no data addressing this possibility.

**Figure 3:**
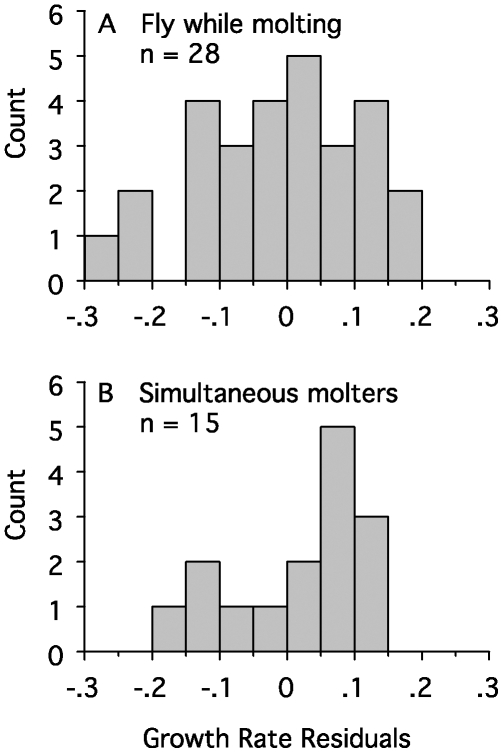
Deviations from the growth rate regression in Figure 1, plotted separately for species that fly while molting and for species that replace their wing quills simultaneously. The latter grow their primaries no slower than birds that fly while molting, suggesting that follicular constraints on the rate of feather synthesis, rather than energetic costs, limit the rate at which flight feathers grow.

We found no evidence that primary growth rate during simultaneous molt is reduced by the energy and nutrient demands of growing all of the flight feathers at once. We divided the 43 species with feather growth rates (Table 1) into two groups, those that replace their primaries simultaneously (*n* = 15 species in two orders) and those that fly while molting (*n* = 28 species in eight orders). We used analysis of covariance (see Methods), with body mass as the covariate to compare feather growth rates between these groups. Remarkably, growth rate did not differ between species with simultaneous primary molt and those that fly while molting (*F* = 1.0; degrees of freedom = 1, 40; *p* = 0.32; Figure 3). Because primary growth rates are similar for birds that grow two or three versus ten primaries simultaneously, primary growth rate seems not to be limited by energy or nutrient demands; others have suggested that growth rate might be limited by follicular-level constraints on the rate at which feathers can be generated [1],[37], and we explore this below.

**Table 1:** Data used to generate the allometric equations of Figure 1A and 1B, and sources for the data on primary growth rate; primary lengths are from museum specimens.

Order	Genus and Species	Primary Growth Rate (mm/d)	Reference	Length of Longest Primary (mm)	Sum of Primary Lengths (mm)	Log_10_ Body Mass (g)	Simultaneous Molt?
**Passeriformes**	*Pica pica*	2.6	[1]	172	1,493	2.250	No
	*Corvus monedula*	3.6	[1]	178	1,482	2.301	No
	*Corvus frugilegus*	3.8	[1]	252	1,991	2.630	No
	*Acrocephalus schoenobaenus*	2.6	[59]	53	454	1.068	No
	*Acrocephalus arundinaceus*	3.4	[59]	74	665	1.479	No
	*Phylloscopus trochilus*	3.1	[59]	54	470	0.949	No
	*Luscinia luscinia*	4.2	[1]	69	598	1.394	No
	*Luscinia svecica*	3.2	[59]	57	522	1.260	No
	*Oenanthe oenanthe*	3.8	[1]	72	622	1.391	No
	*Motacilla alba*	4.5	[1]	67	571	1.255	No
	*Lanius senator*	3.3	[59]	77	626	1.477	No
	*Carpodacus mexicanus*	3.0	[1]	64	565	1.330	No
	*Carduelis flammea*	2.9	[1]	58	497	1.114	No
	*Carduelis chloris*	2.4	[1]	70	591	1.439	No
	*Pyrrhula pyrrhula*	2.4	[1]	73	664	1.474	No
	*Passer domesticus*	2.7	[1]	57	523	1.444	No
**Anseriformes**	*Cygnus cygnus*	9.0	[1]	407	3,409	3.966	Yes
	*Cygnus olor*	6.9	[1]	406	3,485	4.031	Yes
	*Coscoroba coscoroba*	5.0	[1]	329	2,763	3.633	Yes
	*Anser anser*	5.5	[1]	308	2,523	3.651	Yes
	*Chen caerulescens*	7.9	[1]	297	2,400	3.470	Yes
	*Chen rossii*	7.9	[1]	266	2,049	3.201	Yes
	*Branta bernicla*	5.7	[1]	258	1,892	3.114	Yes
	*Branta leucopsis*	7.3	[1]	291	2,243	3.227	Yes
	*Branta canadensis interior*	7.8	[1]	357	2,734	3.585	Yes
	*Anas platyrhychos*	4.5	[55]	198	1,541	3.034	Yes
**Other orders**	*Halcyon leucocephala*	3.6	[1]	78	697	1.628	No
	*Oceanodroma homochroa*	1.7	[1]	106	774	1.567	No
	*Streptopelia roseogrisea*	5.5	[1]	129	1,018	2.170	No
	*Gypaetus barbatus*	6.6	[1]	600	4,869	3.778	No
	*Gyps africanus*	4.4	[1]	440	3,853	3.736	No
	*Phasianus colchicus*	6.1	[1]	181	1,573	3.055	No
	*Gallus gallus*	4.0	[1]	192	1,527	2.932	No
	*Falco tinnunculus*	4.2	[1]	194	1,545	2.277	No
	*Meleagris gallopavo*	7.5	[1]	395	3,289	3.747	No
	*Larus hyperboreus*	8.0	[1]	323	2,588	3.150	No
	*Pandion haliaetus*	7.9	[1]	372	2,714	3.172	No
	*Larus marinus*	9.5	[1]	343	2,760	3.220	No
	*Bugeranus carunculatus*	11.0	[1]	475	4,159	3.900	Yes
	*Grus grus*	9.0	[1]	438	3,645	3.750	Yes
	*Grus vipio*	9.0	[1]	428	3,542	3.750	Yes
	*Grus leucogeranus*	9.0	[1]	450	3,758	3.830	Yes
	*Grus japonensis*	11.0	[1]	478	4,053	3.929	Yes

The last two columns are used to compare the mass-adjusted rate of primary growth for species that fly while molting and species that replace their flight feathers simultaneously.

## Discussion

Our discovery that the time required to replace the primaries (either one by one or all simultaneously) increases disproportionately with body size as *M* ^0.14^ provides a general explanation for much of the variation in primary molt patterns in birds. All birds share the same annual cycle of environmental conditions and seasonal periods available for reproduction, molt, migration, and other activities. The slower feather growth in large species constrains their allocation of time to molt and the degree to which the molt can be completed in a single year. At one extreme, most small temperate species are well known to replace all of their primaries annually, whereas many large species take two or more years to complete their primary molt (Figure 1C). Large species, in which flight feather replacement is typically incomplete, probably grow stronger flight feathers, but we are unaware of data addressing this possibility.

Two special adaptations in the molt sequence typify large birds that fly while molting and that often or always have incomplete molts—stepwise primary replacement and division of the primaries into two molt series (Figure 2C). These modes of primary replacement likely evolved to minimize time conflicts between molt and breeding in large birds and to minimize the increase in wing loading that accompanies reduced primary feather area during molt. Large birds that are rendered flightless by simultaneous flight feather molts always replace all of their primaries annually (Figure 1C), and the modal size for species with simultaneous primary molts most closely approaches the size of the largest flying birds (Figure 2D), implying that simultaneous replacement of the primaries permits size increases by dramatically reducing the time required to replace the flight feathers. Finally, primary growth rate is not depressed in species that grow all their flight feathers simultaneously, suggesting that feather growth rate does not depend on the availability of energy or nutrients. We suggest below that feather growth rate is similarly constrained in sequential and simultaneous molt systems by similar architecture of the growing region at the base of the feather.

That flight feather growth rate increases less rapidly with respect to body mass than does feather length offers a general explanation for the impact of molting on avian life histories. Large species have long reproductive cycles and molt periods, with the result that individuals often replace fewer flight feathers in a molt that follows successful breeding [38],[39]. An individual having a succession of such incomplete molts might accumulate so many worn feathers that its success in subsequent breeding attempts might decline, even to the point of skipping a breeding opportunity to clear over-worn flight feathers from the wing [2]. A large investment in breeding in one year often results in reduced adult survival or reduced breeding success in the following year [40]–[43], but the mechanism underlying this trade-off has been elusive. Accumulated feather wear may well be the culprit, particularly for large species. Even small species with complete molts apparently grow low-quality feathers after heavy investment in breeding [44],[45]. This suggests that feather quality likely links high breeding investment in one year to low success the following year, even though the long post-breeding period available to small temperate-latitude species seems more than adequate for a complete physiological recovery from investment in reproduction. Feathers simply cannot be repaired!

The time constraint on molting in large birds cannot be overcome by growing more feathers simultaneously because of the size-related scaling of the power required for sustained flight and the maximum power available from the flight muscles [28]. For small birds, maximum power is considerably larger than that required for sustained flight, so small birds can fly with large molt gaps in their wings. For large birds, the difference in the power required for sustained flight and the maximum power available is relatively small, making flight with proportionately similar molt gaps impossible. Thus large birds that fly while molting cannot compensate for the relatively slow growth of their primaries by replacing more primaries simultaneously.

The allometric disparity between feather size and feather growth lead us to ask how the 70-kg *Argentavis*, with a wing span of 7 m and outer primaries that were 1,500 mm long [20], almost four times those of the mute swan, could replace its enormous wing quills frequently enough to maintain good flight performance and reproduce. California and Andean Condors have masses of only 10 kg and 12.5 kg, respectively, and California Condors need 2–3 years to replace all of their primaries [17]. *Argentavis* was simply so huge that it might have overcome time constraints on molting by replacing its enormous flight feathers simultaneously, as do the largest geese and swans. Perhaps it did so every 2–3 years by storing sufficient protein in muscles to shelter in caves or cliffs for a simultaneous replacement of the wing quills, which was estimated to require 74 days (Figure 1B). Although no living raptors replace their primaries simultaneously, the evolutionary transition from sequential to simultaneous replacement of the flight feathers might require few changes in the neurophysiological controls that regulate molt; indeed, some individual flamingos and hole-nesting hornbills have been observed change molt patterns, sometimes molting sequentially and retaining flight, and sometimes molting synchronously and becoming flightless [46],[47]. Because basal metabolic rate increases with body size at an allometric coefficient of about 0.72, whereas fat loading increases with an allometric coefficient that is greater than 1.0, long fasts are possible for large species [48]. All living penguins fast while replacing their body plumage on land and use protein stored in their breast muscles to build feathers. In the 35-kg Emperor Penguin *Aptenodytes fosteri*, this fast lasts about 35 days, during which time individuals lose 50% of their body mass [49]. Several fossil penguins, which surely also fasted while molting on land, weighed up to 100 kg [50]–[52]. If penguins can store enough protein and energy to replace their very dense and heavy body plumage while fasting, then our suggestion that *Argentavis* could have replaced its flight feathers from stored reserves seems plausible.

The constraints that feather growth places on molt and other aspects of the annual cycle depend on the positive allometry dictating increasing time required to complete the growth of a single flight feather with respect to increasing body mass. Flight feather growth rate approximates 1/6 power scaling, while flight feather length approximates 1/3 power scaling. Among species of different size that maintain isometric proportions, lengths scale as the 1/3 power of volume. Thus, feather length is dimensionally isometric. Feathers elongate by cell division within a cylinder of collar cells at the base of the growing feather in the follicle, an invagination of the skin [53]. Cell division is followed by cell enlargement, differentiation, and keratinization further along the base of the growing feather and is supplied by blood circulation through the dermal feather pulp within the feather base. The growth zone, within which the barbs of the feather vane also grow, is essentially a linear structure that produces a two-dimensional feather. If the growth zone were to scale in proportion to the length of the grown feather, then the rate of growth would be inversely proportional to the square root (allometric scaling factor 0.5) of feather length. We tested this prediction using a log-log regression of feather growth rate on length of the longest primary, and found the predicted allometric coefficient of 0.5 (*b* = 0.50±0.05 SD, *F* = 86.7, degrees of freedom = 1,41; *p*<0.0001). Other considerations would include the diameter of the follicle, which clearly increases with feather size, but no comparative data are available. If the length of the cylinder of collar cells had a fixed number of rows of dividing cells regardless of feather length, then the growth rate of the two dimensional feather structure would be related to the one-dimensional circumference of the collar, again leading to an 0.5 allometry of growth as a function of size.

The length of the growing region of a feather might be constrained by structural considerations, because the base of the feather, which is filled with a soft dermal pulp within a non-keratinized cylinder of dividing and differentiating epidermal cells, is quite weak. It is not unusual for growing feathers to break at this point. Although further measurements of the growing regions of primary feathers will be required to work out the basis for the square-root allometry between growth rate and feather size, the linear-to-surface relationship that transforms the cylindrical growing region into a two-dimensional feather provides a plausible mechanism at this point for understanding variation in patterns of primary feather molt as a function of body size in birds and how molt might set an upper limit to the size of flying birds.

## Methods

Estimates of primary growth rate (from repeated measures of growing feathers) were obtained from the literature for 43 species of birds (Table 1). For each species, we measured the lengths of the primary flight feathers for one adult male and one adult female using museum specimens, and averaged values for the two sexes in our analyses. For 77 species across a large size range of flying birds, we estimated the fraction of adults that had replaced all their primaries in the previous molt by examining flight feather condition on 20 museum specimens obtained during nonmolting periods. When 20 adults were not available for a species in the collections we examined, fewer specimens were sampled (Table S1, numbers of each species examined). To compare distributions of avian masses with respect to mode of primary replacement, we used the masses for the birds of the world compiled by Dunning [22]. We included races within species if they differed in mass by 10% or more (some differed by more than 100%). We used references [5],[15],[54],[55] and Rohwer (data not shown) to characterize the mode of primary replacement, which, unfortunately, has not been described for several major groups of birds. When mode of primary replacement was assigned using [55] or Rohwer (data not shown), we assumed that all members of a genus followed the molt strategy known for any member of that genus, unless additional data were available or unless body mass variation was too great to safely generalize.

To analyze the relationship between the mode of primary replacement and body size, we divided the complexities of primary replacement across birds into three basic modes, Simple, Complex, and Simultaneous [15]. Some cuckoos and kingfishers do not fit these categories and were omitted [5]. In species with Simple primary replacement, molt begins at innermost P1 and proceeds distally until P9 or P10 is replaced. All species in this category feature a single wave of feather replacement, but they often lose adjacent feathers in quick succession, resulting in large gaps in their wings. Complex primary replacement occurs in two ways, one or the other of which usually characterizes large species that maintain the ability to fly while molting. In the first, called stepwise molting, the primaries constitute a single molt series [56], but several waves of feather replacement progress through the primaries simultaneously in adults [5],[32],[37],[57]. In the second, the primaries are organized into two separately activated and nonoverlapping molt series; this mode of replacement generates two waves of growing primaries if both series are activated during a single episode of molting [5],[16],[58]. The third mode of primary replacement is Simultaneous, whereby all primaries (and, usually, all secondaries) are lost and re-grown more or less simultaneously, resulting in a 3–6-week period of flightlessness.

Allometric relationships between feather length, feather growth rate, and body mass were determined by regression and analysis of covariance of log-transformed values based on type III sums of squares, in which taxonomic orders (Anseriformes [*n* = 10], Passeriformes [16], Coraciiformes [1], Procellariiformes [1], Columbiformes [1], Falconiformes [4], Galliformes [3], Charadriiformes [2], and Gruiformes [5]) were entered as a main effect to avoid fortuitous relationships resulting from heterogeneity among taxa. Interactions between taxa and the independent variable were not significant and were dropped from the models. Because the regression slopes of models with taxon as a main effect did not differ from those obtained from simple regressions, we report here the slopes of the simple regressions (41 error degrees of freedom in each case): longest primary feather versus body mass, *b* = 0.325±0.010, *p*<0.0001, *R* ^2^ = 0.961; sum of primary lengths versus body mass, *b* = 0.316±0.009, *p*<0.0001, *R* ^2^ = 0.965; growth rate of primary versus body mass, *b* = 0.171±0.017, *p*<0.0001, *R* ^2^ = 0.713. Analyses were carried out with the GLM procedure of the Statistical Analysis System version 9.1 (SAS Institute).

## Supporting Information

Table S1 Species and numbers of adults used to assess completeness of molt for Figure 1C.
(0.18 MB DOC)
