# Supplementary material for: Allometry of the Duration of Flight Feather Molt in Birds
Source: PLoS Biol. 2009 Jun 16;7(6):e1000132. doi: 10.1371/journal.pbio.1000132 (PMC2690433; doi:10.1371/journal.pbio.1000132)
Supplement: Table S1 — Species and numbers of adults used to assess completeness of molt for Figure 1C. (0.18 MB DOC) [file pbio.1000132.s001.doc]

**Supporting Information**

Table S1. Species and numbers of adults used to assess completeness of molt for Figure 1C.

| Species | Percent complete | Simultaneous molt? | Mass (g) | N |
| --- | --- | --- | --- | --- |
| Gavia stellata | 100 | Yes | 1551.0 | 19 |
| Gavia pacifica | 100 | Yes | 2350.0 | 20 |
| Podiceps auritus | 100 | Yes | 453.0 | 20 |
| Podiceps grisegena | 100 | Yes | 1023.0 | 20 |
| Diomedea exulans | 0 | No | 9500.0 | 20 |
| Diomedea epomophora | 0 | No | 10500.0 | 20 |
| Phoebastria nigripes | 0 | No | 3148.0 | 20 |
| Puffinus bulleri | 40 | No | 380.0 | 20 |
| Oceanodroma furcata | 100 | No | 55.3 | 20 |
| Pelecanus onocrotalus | 0 | No | 15000.0 | 20 |
| Pelecanus crispus | 0 | No | 13000.0 | 15 |
| Pelecanus erythrorhynchos | 0 | No | 7500.0 | 20 |
| Phalacrocorax auritus | 15 | No | 1679.0 | 20 |
| Ardea herodias | 0 | No | 2390.0 | 20 |
| Ardea goliath | 0 | No | 9500.0 | 12 |
| Nycticorax nycticorax | 20 | No | 883.0 | 20 |
| Mycteria americana | 25 | No | 2500.0 | 20 |
| Ciconia ciconia | 0 | No | 4000.0 | 20 |
| Ephippiorhynchynchus asiaticus | 0 | **No** | **6000.0** | **7** |
| Jabiru mycteria | 0 | No | 8000.0 | 13 |
| Leptoptilos crumeniferus | 0 | No | 9000.0 | 17 |
| Cygnus buccinator | 100 | Yes | 10650.0 | 20 |
| Anas crecca | 100 | Yes | 341.0 | 20 |
| Anas platyrhynchos | 100 | Yes | 1082.0 | 20 |
| Somateria spectabilis | 100 | Yes | 1617.5 | 20 |
| Histrionicus histrionicus | 100 | Yes | 622.5 | 20 |
| Clangula hyemalis | 100 | Yes | 873.0 | 20 |
| Melanitta nigra | 100 | Yes | 950.0 | 20 |
| Chauna torquata | 0 | No | 4000.0 | 14 |
| Coragyps atratus | 0 | No | 7500.0 | 12 |
| Gymnogyps californianus | 0 | No | 12750.0 | 15 |
| Vultur gryphus | 0 | No | 14000.0 | 16 |
| Pandion haliaetus | 0 | No | 8500.0 | 20 |
| Haliaeetus leucocephalus | 0 | No | 4683.5 | 20 |
| Haliaeetus pelagicus | 0 | No | 8500.0 | 14 |
| Gypaetus barbatus | 0 | No | 11000.0 | 20 |
| Gyps fulvus | 0 | No | 9500.0 | 20 |
| Aegypius monachus | 0 | No | 12500.0 | 11 |
| Accipiter striatus | 100 | No | 138.5 | 20 |
| Buteo jamaicensis | 25 | No | 1126.0 | 20 |
| Harpia harpyja | 0 | No | 9000.0 | 8 |
| Aquila chrysaetos | 0 | No | 6665.0 | 20 |
| Sagittarius serpentarius | 0 | No | 5000.0 | 12 |
| Caracara plancus | 100 | No | 950.0 | 20 |
| Megapodius freycinet | 65 | No | 600.0 | 20 |
| Alectura lathami | 53 | No | 2300.0 | 14 |
| Crax rubra | 100 | No | 4000.0 | 15 |
| Perdix perdix | 100 | No | 389.5 | 20 |
| Coturnix coturnix | 100 | No | 30.0 | 20 |
| Bonasa bonasia | 100 | No | 395.0 | 20 |
| Meleagris gallopavo | 100 | No | 7000.0 | 20 |
| Rallus elegans | 100 | No | 355.0 | 20 |
| Gallinula chloropus | 100 | No | 305.0 | 20 |
| Fulica americana | 100 | No | 645.0 | 20 |
| Grus canadensis | 0 | No | 4375.0 | 20 |
| Choriotis kori | 0 | No | 19000.0 | 20 |
| Pluvialis squatarola | 100 | No | 250.0 | 20 |
| Haematopus ostralegus | 100 | No | 575.0 | 20 |
| Numenius phaeopus | 100 | No | 450.0 | 20 |
| Numenius americanus | 100 | No | 650.0 | 20 |
| Philomachus pugnax | 100 | No | 175.0 | 20 |
| Stercorarius parasiticus | 100 | No | 440.0 | 20 |
| Stercorarius longicaudus | 100 | No | 275.0 | 20 |
| Larus glaucescens | 100 | No | 1010.0 | 20 |
| Larus marinus | 100 | No | 1600.0 | 20 |
| Uria aalge | 100 | Yes | 992.5 | 20 |
| Cepphus columba | 100 | Yes | 487.0 | 20 |
| Brachyramphus marmoratus | 100 | Yes | 222.0 | 20 |
| Brachyramphus brevirostris | 100 | Yes | 224.0 | 20 |
| Synthliboramphus antiquus | 100 | Yes | 206.0 | 20 |
| Fratercula corniculata | 100 | Yes | 619.0 | 20 |
| Tyto alba | 0 | No | 466.0 | 20 |
| Otus kennicottii | 25 | No | 187.0 | 20 |
| Bubo virginianus | 30 | No | 1407.5 | 20 |
| Strix varia | 0 | No | 716.5 | 20 |
| Aegolius acadicus | 60 | No | 82.9 | 20 |
| Alcedo atthis | 90 | No | 32.3 | 20 |
| Halcyon chloris | 100 | No | 73.8 | 20 |
| Cyanocitta stelleri | 100 | No | 106.0 | 20 |
| Pica pica | 80 | No | 177.5 | 20 |
| Corvus brachyrhynchos | 100 | No | 448.0 | 20 |
| Corvus corax | 100 | No | 1097.0 | 20 |
| Parus montanus | 100 | No | 10.8 | 20 |
| Parus ater | 100 | No | 9.5 | 20 |
| Parus major | 100 | No | 18.3 | 20 |
| Psaltriparus minimus | 100 | No | 5.3 | 20 |
| Regulus satrapa | 100 | No | 6.2 | 20 |
| Luscinia calliope | 100 | No | 25.4 | 20 |
| Turdus migratorius | 100 | No | 77.3 | 20 |
| Dendroica townsendi | 100 | No | 8.9 | 20 |
| Xanthocephalus xanthocephalus | 100 | No | 64.5 | 20 |
| Molothrus aeneus | 100 | No | 62.0 | 20 |
| Molothrus ater | 100 | No | 43.9 | 20 |
| Icterus bullockii | 100 | No | 33.6 | 20 |
